# MST1/2 Balance Immune Activation and Tolerance by Orchestrating Adhesion, Transcription, and Organelle Dynamics in Lymphocytes

**DOI:** 10.3389/fimmu.2020.00733

**Published:** 2020-05-06

**Authors:** Yoshihiro Ueda, Naoyuki Kondo, Tatsuo Kinashi

**Affiliations:** Department of Molecular Genetics, Institute of Biomedical Science, Kansai Medical University, Hirakata, Japan

**Keywords:** Mst1/2, lymphocyte trafficking, effector differentiation, cell polarity and adhesion, integrin, vesicle transport

## Abstract

The STE20-like serine/threonine kinases MST1 and MST2 (MST1/2) are mammalian homologs of Hippo in flies. MST1/2 regulate organ size by suppressing the transcription factor YAP, which promotes proliferation. MST1 is predominantly expressed in immune cells, where it plays distinct roles. Here, we review the functions of MST1/2 in immune cells, uncovered by a series of recent studies, and discuss the connection between MST1/2 function and immune responses. MST1/2 regulate lymphocyte development, trafficking, survival, and antigen recognition by naive T cells. MST1/2 also regulate the function of regulatory T cells and effector T cell differentiation, thus acting to balance immune activation and tolerance. Interestingly, MST1/2 elicit these functions not by the “canonical” Hippo pathway, but by the non-canonical Hippo pathway or alternative pathways. In these pathways, MST1/2 regulates cellular processes relating to immune response, such as chemotaxis, cell adhesion, immunological synapse, gene transcriptions. Recent advances in our understanding of the molecular mechanisms of these processes have revealed important roles of MST1/2 in regulating cytoskeleton remodeling, integrin activation, and vesicular transport in lymphocytes. We discuss the significance of the MST1/2 signaling in lymphocytes in the regulation of organelle dynamics.

## Introduction

The serine/threonine kinases MST1 (STK4) and MST2 (STK3) belong to the mammalian STE20-like kinase family. The *Drosophila* homolog of MST1 and MST2 (MST1/2), Hippo (HPO), is the core enzyme of a pathway that controls organ size by regulating cell proliferation and differentiation (1–4). In the canonical Hippo signaling pathway of *Drosophila*, HPO, complexed with Salvador (SAV), phosphorylates and activates Nuclear Dbf-2-related (NDR) family kinase Warts (WTS) and its adaptor Mob as Tumor Suppressor (MATS), which are orthologous to mammalian LATS1/2 and MOB1A/B, respectively. A *Drosophila* ortholog of YAP, YKI, is a transcriptional activator to promote proliferation by collaborating with co-activators. WTS phosphorylates YKI to inhibit its function. In the non-canonical Hippo pathway of *Drosophila*, TRC, an ortholog of mammalian NDR, also acts as a downstream kinase of HPO and plays roles in the morphogenesis of epithelial cells and in dendritic tiling and maintenance of neural cells (5–7).

In the canonical Hippo pathways in mammals, MST1/2 activate MOB1A/B and LATS1/2. LATS1/2 phosphorylate and inhibit transcriptional activity of YAP/TAZ which promotes gene transcription related to survival and proliferation. Thus, the canonical pathway is important for tissue development and regeneration (8, 9). In addition to the canonical pathway, NDR1/2 are also phosphorylated by MST1 (10) and activate the non-canonical Hippo pathways that regulate various cellular processes (11–13). MST1/2 also phosphorylate other proteins to control their functions as alternative pathways.

It has been increasingly recognized that MST1/2 are involved in innate and adaptive immune regulation in mammals. Homozygous nonsense mutations of *MST1* in humans induce a combined immunodeficiency with severe lymphopenia, neutropenia, and hypergammaglobinemia characterized by recurrent infection (14–17). Some *MST1*-null patients develop autoimmune cytopenias and disseminated EBV viremia. The combined phenotypes of immunodeficiency and autoimmunity are recapitulated by mouse models deficient for MST1 alone, or MST1/2 in a T cell–specific manner (18–23). These mice exhibit hypoplastic lymphoid tissues and develop autoimmune phenotypes with age. In both humans and mice, MST1/2-deficient lymphocytes have defects in chemokine-induced migration and integrin-dependent adhesion, as well as elevated rates of apoptosis. Furthermore, MST1/2 also control effector T cell differentiation by modulating transcription factors important for this process. In the first section, we describe the detailed roles of MST1/2 in lymphocyte development, trafficking, tolerance, survival, and effector differentiation, and discuss the balance of activation and tolerance of lymphocyte controlled by MST1/2.

How do MST1/2 regulate the balance mechanistically? Recent studies have shed light on the downstream signals of MST1/2 that regulate lymphocyte trafficking and antigen-recognition. MST1/2 promote integrin activation through the non-canonical Hippo pathway via NDR1/2. MST1 also regulates F-actin dynamics in response to chemokines by regulating Rho family GTPase and L-plastin. Furthermore, MST1/2 play a vital role in antigen recognition of T cells by regulating formation of the immunological synapse (IS), the T cell interface for antigen recognition. Analysis of IS in MST1/2-deficient T cells has revealed the important role of MST1/2 signaling in the regulation of vesicular trafficking. In the second section, we summarize the MST1/2-mediated signals that direct integrin activation, F-actin dynamics, and vesicular trafficking.

## Part I: the Function of MST1/2 in Lymphocyte Regulation

A major role of MST1/2 is the regulation of integrin-mediated cell adhesion and cell polarity in response to chemokine or antigen-stimulation, resulting in controlling lymphocyte development and trafficking. Other roles of MST1/2 are the regulations of lymphocyte survival and the effector T cell differentiation via modulating activity and stability of transcription factors. We describe the regulatory processes in detail below.

## MST1/2 Regulate Lymphocyte Development and Trafficking by Integrin-Dependent Adhesion

Several studies of MST1-deficient and MST1/2-double deficient mice reveal that MST1/2 are important for the development and trafficking of lymphocytes by facilitating processes mediated by chemokine and integrin.

Homing of hematopoietic stem cells (HSCs) to bone marrow (BM) requires expression of chemokine receptor CXCR4 (24) and integrins α4β1(VLA-4), α4β7, and α6β1 (25). MST1/2 are required for homing of HSCs and T-cell progenitors. MST1-deficient or MST1/2-double deficient HSCs fail to migrate into BM and are unable to reconstitute all types of hematopoietic-lineage cells (26).

**FIGURE 1:**
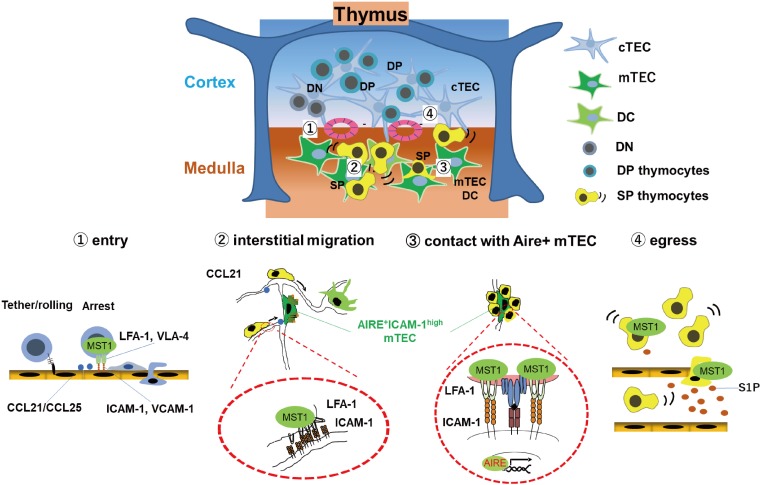
MST1/2 are required for thymocyte trafficking and antigen recognition. MST1 is required for several processes of thymocyte trafficking: (1) Homing of T cell progenitors to the thymus via integrin-dependent adhesion on the vessels; Self-antigen scanning driven by promoting (2) rapid interstitial migration and (3) arrest on mTEC-expressing self-antigens within the medulla via LFA-1/ICAM-1 interaction. (4) Egress of mature thymocytes via vessels or lymphatics in response to chemokine CCL21 or S1P.

The chemokine receptors CCR7/CCR9, as well as integrins LFA-1/VLA-4 and their counter-receptors ICAM-1/VCAM-1, are required for efficient entry of T cell progenitors into the thymus (27, 28). In support, the integrin coactivator Kindlin-3 is required for T cell progenitor homing (29). Similar to MST1/MST2-deficient HSC, MST1/2 double deficient T cell progenitors have defects in migration into the thymus (26), suggesting that MST1/2 play a role in regulating integrins during this process. MST1/2 are also involved in negative selection of autoreactive thymocytes, as well as egress from the thymus (Figure 1). After entry into the thymus, successful TCR rearrangement facilitates differentiation of T cell progenitors into CD4 + CD8 + double-positive (DP) thymocytes, which move from the cortex to the medulla and differentiate into either CD4 or CD8 single-positive (SP) cells (30). In the medulla, medullary epithelial cells (mTECs) express organ-specific self-antigens via transcription factor AIRE (31). Dendritic cells (DCs) also present self-antigens expressing by themselves or received from mTEC. During this process, SP cells randomly migrate within the medulla and interact with *Aire*^+^mTECs and DCs (18, 32). Strong interactions of autoreactive SP cells with self-antigen on *Aire*^+^mTECs or DCs trigger an activation of SP cells and induce cell death by negative selection (30). Otherwise, autoreactive SP cells express both IL-2 receptor and FOXP3 in response to self-antigen, followed by differentiation of SP cells to regulatory T cells (Tregs). FOXP3 is a master transcription factor required for Treg differentiation and maintenance.

MST1-deficient SP cells have an intrinsic defect in integrin-dependent migration within the medulla, as well as contact with *Aire*^+^mTECs expressing self-antigen, resulting in inefficient antigen scanning (18). Inefficient recognition of self-antigen decreases frequency of activated cells or attenuates activation of TCR signals in autoreactive MST1-deficient SP cells, thereby causing defective negative selection of autoreactive T cells and reduction of Tregs, facilitating autoimmune phenotypes of MST1-deficient mice (18).

After selection, SP thymocytes egress from the thymus via blood or lymphatic vessels and emigrate to secondary lymphoid organs. This process requires chemotactic migration in response to sphingosine-1-phosphate (S1P) or CCL21. Deficiency of MST1 or MST1/2 impairs egress of mature thymocytes due to a defect in chemotactic migration toward S1P and CCL19/21 (19, 33).

Naïve T cells are less abundant in spleen and lymph nodes of MST1-deficient or MST1/2-double deficient mice due to impaired integrin-mediated trafficking (21, 22). MST1/2-deficient T cells fail to stably attach high endothelial venules (HEVs) and efficiently transmigrate into lymph nodes (21, 34). Consistent with this, MST1/2-deficient T cells have a defect in flow-resistant adhesion to endothelial integrin ligands such as ICAM-1 and VCAM-1 upon chemokine stimulation. MST1 is also involved in cell polarization in response to chemokines. MST1 deficiency in T cells causes impaired interstitial migration within lymph nodes due to the defects in integrin-mediated adhesion and cell polarization (21).

MST1/2 are also important for late B cell production. Mature B cells have three subsets: follicular (FO) B-2, marginal zone (MZ) B, and B-1a B cells. FO B-2 cells are responsible for T cell–dependent antibody responses, whereas MZ B cells are localized at the splenic marginal zone and are important for T cell–independent early antibody production against blood-borne antigens. B-1a B cells are involved in the production of natural antibodies (35). In mice deficient for MST1 or MST1/2, MZ B cells and B-1a B cells are less abundant in the spleen, with modest effects on early B cell development in the BM (36). Retention and survival of MZ B cells in the spleen are dependent on chemokines such as S1P and CXCL12 (37, 38) and the integrin ligands ICAM-1 and VCAM-1 (39). Similarly, the number of BM recirculating B cells, of which homing is also dependent on integrin signals (40), is reduced in the BM (36). Severe loss of FO B-2 cells in lymph nodes of MST1-deficient or MST1/2-deficient mice is also observed, due to defective migration into lymph nodes via high endothelial venules (HEVs) (21, 22, 36). These processes are highly dependent on CCL21/CXCL12 and integrin (41), indicating critical roles for MST1/2 in integrin-mediated adhesion during late B cell development.

## The Pivotal Roles of MST1/2 in Antigen Recognition by Regulating Integrin-Dependent Cell-Cell Contacts

Naïve T cells recognize cognate antigen on major histocompatibility complex (MHC) presented by antigen-presenting cells such as DCs, and then become activated, proliferate, and differentiate into memory or effector cells. Initial studies examined the proliferative response to direct activation of TCR crosslinking by antibodies in MST1-deficient mice (21, 22, 42). More recent work examined the roles of MST1/2 in antigen-specific proliferation. Upon antigen-specific stimulation, MST1- or MST1/2-deficient T cells fail to form stable contacts with DCs in both *in vitro* and within lymph nodes (34). As a result, MST1- or MST1/2-deficient T cells exhibit defective proliferation in response to antigen stimulation (34). These defects are likely due to defective adhesion mediated by LFA-1 and ICAM-1. Moreover, MST1-deficient T cells are not able to form pSMAC (LFA-1/ICAM-1 cluster) or cSMAC (TCR/pMHC cluster) in the IS on lipid bilayers presenting peptide/MHC and ICAM-1 (34) (see section II). Thus, MST1/2 play an essential role in forming the adhesion structure required for antigen recognition of T cells.

Furthermore, important roles of MST1 for antigen recognition are emphasized by requirement of MST1 in contact-dependent suppressor functions of Tregs (43, 44). Inhibition of T cell proliferation by MST1-deficient Tregs is comparable to that of wild-type T cells when anti-CD3 antibodies are used for stimulation (43). However, MST1-deficient Tregs do not efficiently inhibit the proliferation of naïve T cells in response to antigen presented on DCs and also do not prevent experimental colitis by adoptive transfer of naïve T cells into severely immunodeficient mice. The absence of MST1 in Tregs decreases cognate interactions with DCs, resulting in inefficient downregulation of the costimulatory molecule CD86 in DCs, indicating that antigen-specific Treg suppression requires LFA-1–mediated contact with DCs. These defective functions of Treg are considered to be associated with autoimmune phenotype of MST1-deficeint mice.

## MST1/2 Regulate the Differentiation of Effector T Cell Subsets by Regulating Transcriptional Factors

**FIGURE 2:**
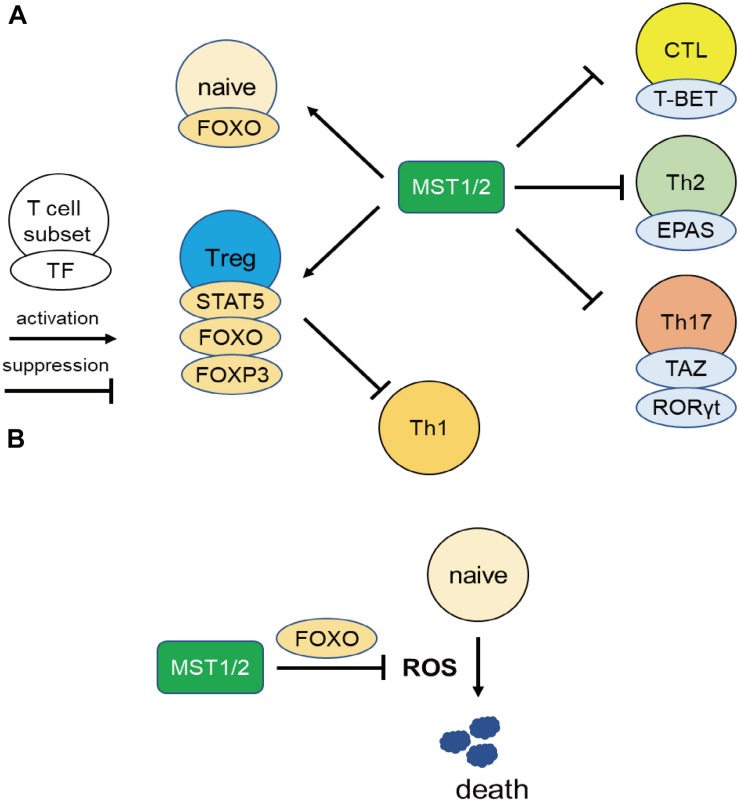
MST1 regulate T cell survival and differentiation via regulating transcriptional activity. **(A)** MST1/2 positively regulate Treg differentiation or functions through STAT5, FOXO, and FOXP3. Treg also suppress Th1 responses. On the other hand, MST1/2 inhibit the differentiation or functions of CTL, Th2, and Th17 cells via negative regulation of transcription factors T-BET, EPAS, and RORγt. **(B)** MST1/2 promote FOXO-mediated regulation against oxidative stress in naïve T cells.

Series of resent works uncovered the integrin-independent regulation of MST1/2, especially in the effector differentiation and functions via regulation of transcriptional factors, and are described below from the point of view of the regulation of gene transcription (Figure 2A).

Several studies have shown that MST1 is important for generation, maintenance, and function of Treg by regulating FOXP3 expression in Tregs. The transcription factor FOXO binds to the *Foxp3* promoter and promotes its transcription. Consistent with this, FOXO1/3-deficient mice have reduced numbers of Tregs (45, 46). MST1 activates FOXO1/3, resulting in enhancement of *Foxp3* transcription in Tregs (23). A deacetylase SIRT1 is known to deacetylate FOXP3 and promotes proteasomal degradation of FOXP3 (47). MST1 prevents FOXP3 degradation in Tregs by inhibiting SIRT1-mediated deacetylation of FOXP3 by phosphorylating SIRT1 (48, 49).

MST1/2 are also involved in the regulation of IL-2R signaling in Tregs. In mice, in which *Mst1/2* were is Treg-specifically mutated, Treg number is not altered at 1 month of age, but decreases significantly with age in peripheral lymphoid tissues, resulting in Th1-associated lethal autoimmune diseases (50). Thus, MST1/2 are required for the maintenance of Treg pools. Mechanistically, MST1/2 positively regulate STAT5 phosphorylation upon IL-2 stimulation and control survival in Tregs. MST1/2 are also required for migration of Treg to T cell zones via the Rho-GTPase RAC1, and enable Treg to access the source of IL-2–producing cells. Downregulation of IL-2 receptor α chain (CD25) in MST1- or MST1/2-deficient Tregs (43, 50) may also contribute to attenuation of IL-2 receptor signaling (43, 50).

MST1/2 deficiency also affects the differentiation of cytotoxic T cells (CTL). During the generation of CTL *in vitro*, lack of MST1 decreases the levels of FOXO in CD8 T cells (51). MST1-deficient CD8 T cells exhibit higher expression of T-bet transcription factors, which is associated with higher expression levels of IFNγ and granzyme B. Consistent with this, MST1-deficient CTLs have greater tumoricidal activity *in vitro*, and suppress tumor progression in mouse models more efficiently, than wild-type CTLs. Thus, MST1 exerts an inhibitory effect on differentiation and function of CTL. On the other hand, YAP, the transcription factor of the canonical Hippo pathway, promotes CTL differentiation (52), but no direct connection between MST1 and YAP in CTLs has yet been demonstrated (53).

MST1/2 may regulate the balance of differentiation of CD4 T cells into Th17 or Treg cells via TAZ, a coactivator of TEAD transcription factors involved in the canonical Hippo pathway (54). Th17 cells express the highest levels of TAZ among the helper T cell subsets. Deletion of TAZ in activated T cells results in reduced abundance of Th17 cells with a reciprocal increase in Tregs. Conversely, overexpression of TAZ increases Th17 abundance at the expense of Tregs. TAZ acts as a co-activator of RORγt, a master regulator of Th17 differentiation, to promote Th17 generation, whereas it inhibits FOXP3 functions by decreasing acetylation mediated by the histone acetyltransferase Tip60. TEAD1 and sequestration of TAZ from RORγt and FOXP3 result in Treg differentiation. Thus, TAZ controls the balance of Th17 and Treg differentiation.

MST1 negatively regulates follicular T helper (Tfh) cell expansion. Tfh cell is a T cell subset to provide survival signals for B cells via cognate interaction, and promote their antibody production in germinal center (GC). In Mst1-deficient mice, Tfh cells are more abundant, and serum levels of antibodies and autoantibodies are elevated (55). Moreover, MST1-deficient Tfh cells express higher levels of IL-21, IL-4, and surface CD40L. The abnormally activated Tfh cells cause aberrant B cell activation in GC and accelerate the differentiation of short-lived plasmacytes.

MST1 also regulates IL-31 production in Th2 cells (56). DOCK8-deficient mice overproduce IL-31 in their Th2 cells and develop atopic skin diseases. In Th2 cells, DOCK8 forms a complex with MST1 and inhibits nuclear translocation of the transcription factor EPAS, independent of the guanine exchange factor activity of DOCK8. Given that EPAS is critical for IL-31 gene expression, this implies that the MST1–DOCK8 axis inhibits IL-31 production. Taken together, these findings demonstrate that MST1/2 restrict effector T cell differentiation by modulating transcription factors.

## MST1 Regulates T Cell Survivals by Attenuating Oxidative Stress

**FIGURE 3:**
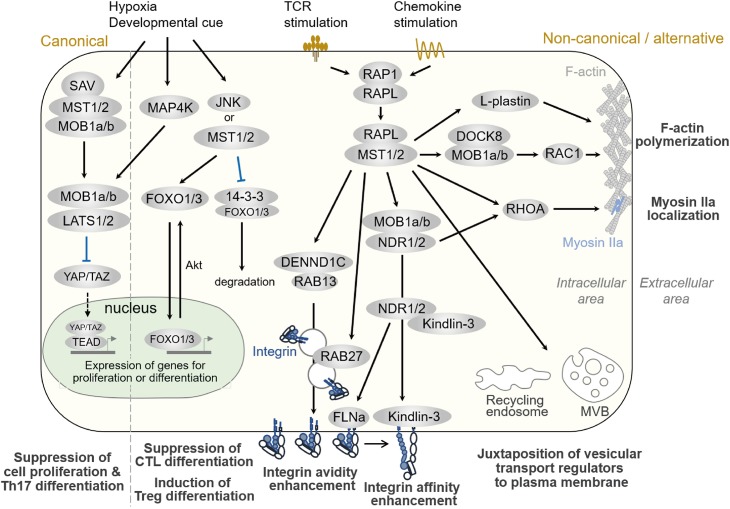
Hippo pathways in lymphocytes. Signaling pathways regulated by MST1/2 in lymphocytes. Compartmentalized areas colored light yellow and light green indicate the intracellular/cytoplasmic area and nucleus, respectively. The canonical Hippo pathway is depicted at the very left, whereas non-canonical/alternative pathways are depicted in the rest of the figure. MVB: multi-vesicular body.

MST1 also promotes T cell survival by protecting from oxidative stress in integrin-independent manner (Figure 2B). Choi et al. reported elevated rates of lymphocyte apoptosis in MST1-deficient mice (20). This is presumably due to increases in the levels of reactive oxygen species (ROS) resulting from downregulation of Sod2 and catalase, exacerbating lymphopenia (20). Under oxidative stress, MST1 phosphorylates FOXO1/3a at Ser212 or Ser207 and disrupts the interaction of FOXO1/3 with 14-3-3 proteins, which promote nuclear translocation and transcriptional activity of FOXO1/3 (20, 57). On the other hand, AKT phosphorylates FOXO1/3a at sites distinct from those targeted by MST1/2, triggering the nuclear export of the transcription factor. Therefore, AKT and MST1 can be thought as the “brake” and “acceleration” pedals for FOXO 1/3 activity, respectively (Figure 3). FOXO1/3 promote transcriptional activation of *Sod2*, catalase, and *Bcl-2* (58). Thus, MST1/2 regulate integrin-dependent trafficking of naïve T cells and protect them from cell death mediated by oxidative stress. The role of MST1/2 in the protection from oxidative stress is also reported in macrophage by stabilizing nuclear factor (erythroid-derived 2)-like 2 (Nrf2).

## MST1/2 as Balancers of Immune Activation and Tolerance in Lymphocytes

In summary, MST1/2 play important roles in adaptive immune regulation. MST1/2 maintains naïve T cell and B cell pools in lymphoid systems by regulating integrin-dependent adhesion and protecting against cell death from oxidative stress (Figure 2). Moreover, MST1/2 promote Treg differentiation and functions, but inhibit effector T cell differentiation. MST1/2 regulate the differentiation of Tregs and immune suppression by these cells through integrin signaling. The importance of integrins in Tregs is consistent with studies showing that a defect in integrin and its activator Talin1 impairs Treg generation and functions (44, 59–61), whereas the constitutively active mutant of the Rap1 small GTPase (RAP1), a master regulator of integrin activation, reciprocally increases Treg abundance (62). MST1/2 also positively regulates FOXP3 expression and IL-2 signaling, contributing to Treg differentiation and functions. MST1/2 also act as a rheostat for effector T cell differentiation by negatively regulating transcription factors important for effector differentiation, such as T-BET, EPAS, and RORγt. Thus, MST1/2 increase the threshold of immune activation and prevent from excessive responses such as autoimmune disease or allergy. Collectively, MST1/2 serve to balance immune activation and tolerance in lymphocytes.

## Part II. Downstream Signaling of MST1/2 in Lymphocyte Regulation

As described in Part I, MST1/2 regulate lymphocyte functions, such as cell trafficking, antigen-recognition, proliferation, and differentiation. Although the canonical Hippo pathway regulates Th17 and Treg differentiation and functions (54), a direct connection from MST1/2 to LATS, TAZ, and YAP during differentiation has not been elucidated. Rather, some studies have reported that factors in the canonical pathway are dispensable in immune cells (33, 63). Since the regulations of integrin activation and cell polarity formation are major pathway to exert these functions in both humans and mice, we focus to introduce the MST1/2 signaling in the non-canonical pathways and alternative pathways for the regulation of integrin activation and cell polarity formation, and discuss the relevance of Mst1/2 signals to F-actin dynamics and vesicular transport machinery in this part (Figure 3).

## Regulation of Cell Adhesion and Polarity by the Non-Canonical Hippo Pathway in Immune Cells

The importance of the non-canonical pathways in MST1 function in lymphocytes has been highlighted by the finding that MST1 is involved in integrin activation in lymphocytes (21, 22). Indeed, MST1-deficient T cells fail to adhere to ICAM-1 and VCAM-1, resulting in impaired arrest on HEVs and defective adhesion to APC (21, 22). In mechanistic terms as shown in Figure 3, upon stimulation by TCR-crosslinking or chemokine, RAP1 is activated and forms a complex with RAPL (64, 65). RAP1/RAPL subsequently binds to MST1. Hetero-dimerization of MST1/RAPL via the SARAH domain regulates MST1 localization and activation at the membrane (65). RIAM also interacts with RAP1 via the RA-PH domain (66), and is thought to form multi-component complex with MST1 and Talin1 (67). Together with ADAP/SKAP1 (68), RAP1/RAPL/MST1 complexes bind to the cytoplasmic tail of αL integrin (67). Deficiency of RAPL or RIAM also causes impaired LFA-1–dependent adhesion (34, 64, 69, 70). Although the binding of Kindlin-3 and Talin1 at the β-integrin cytoplasmic tail regulates integrin activation and clustering, the molecular link between RAP1 and Talin1/Kindlin-3 remains elusive. Recently, RAP1 was shown to bind Talin1 via the F0 and F1 domains and promote localization of Talin1 at the plasma membrane (71–73). In complex with RAPL, MST1 phosphorylates NDR1/2 kinases, family members of LATS1/LATS2 and co-activators of MOB1 (34), upon TCR crosslinking. Phosphorylated active NDR directly binds to Kindlin-3, leading to its recruitment to the contact surface, which is required for the high-affinity binding of LFA-1. NDR2 also phosphorylates Filamin A to facilitate binding of Talin1 and Kindlin-3 to the β2 cytoplasmic tail (74). Thus, NDR1/2 kinases mediate integrin activation through Kindlin-3 and Talin1. In support of these notions, the deficiency phenotypes of MOB1 or NDR1/2 reveal their critical roles in T cell homeostasis as downstream effectors of MST1/2 (63, 75): T cell–specific deletion of *Ndr1/2* or *Mob1a/Mob1b* results in phenotypes similar to those of *Mst1*-null mutation in T cells (63).

Formation of cell polarity is associated with lymphocyte migration, which is typically characterized as F-actin rich lamellipodia at the front and constricted cell bodies, termed uropods, at the rear. Regulation of cell polarity is important for efficient migration of lymphocytes. It is well established that the spatio-temporal activation of RAC and RHOA regulates F-actin development at the front and actomyosin-mediated contraction at the rear. However, the molecular networks that coordinate this process have not been fully elucidated. As depicted in Figure 3, RAP1 signaling to RAPL and MST1/2 is required for lymphocyte cell shape changes upon chemokine stimulation (65, 76). MST1/2- and NDR1/2-deficient T cells exhibit defective polarity in response to CCL19/21, CXCL12, or S1P, concomitant with reduced activation of RAC and RHOA (33, 63). Thus, both MST1/2 and NDR1/2 are involved in lymphocyte polarity through activation of RAC and RHOA.

As for F-actin regulation, MST1 forms a complex with DOCK8 and promotes RAC1 activation in response to CCL19 and S1P in thymocytes or Tregs (33, 50). Introduction of a constitutively active *Rac1G12V* mutant can rescue the MST1/2-deficient phenotype in Treg (33, 50), suggesting an important role for MST1 in RAC regulation. Recently, MST1 was shown to phosphorylate the actin-bundling protein L-plastin (LPL) at T98 and regulate turnover of F-actin and lamellipodia formation (77). Mice deficient for LPL have phenotypes similar to those of the mice deficient for MST1, including the defects in cell polarization, lamellipodial formation, and cell migration. These results indicate that MST1/2 is important for F-actin dynamism via regulation of RAC.

RHOA is also important for this process. RHOA activates ROCK to promote uropod formation via activation of Myosin light chain 2 and redistribution of Ezrin/Radixin/Moesin (ERM) proteins (78). MST1/2 also regulate uropod formation via RHOA activation (33) and assembly of Myosin IIa (79). In addition, NDR1/2 are involved in RHOA activation, as NDR1/2-deficient T cells exhibit a decrease in RHOA activation upon chemokine stimulation (63). Further detailed analyses will be required to uncover the relevance of RHOA to the Hippo pathway.

## MST1/2 as Organizer of Membrane Trafficking in the Immunological Synapse

**FIGURE 4:**
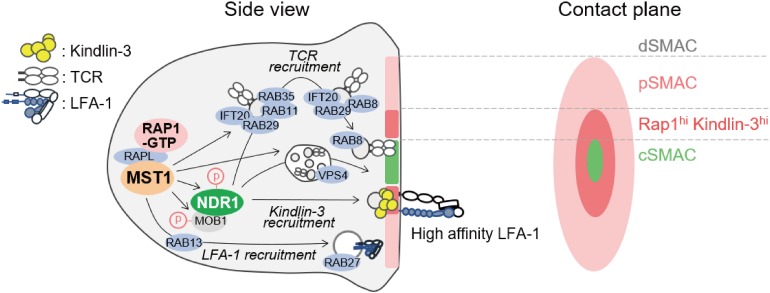
Model of regulation of immunological synapse (IS) maturation by non-canonical Hippo signaling. Regulators of immunological synapse maturation related to the non-canonical Hippo signaling. The IS is formed at the T cell interface with APC. The mature IS contains central supramolecular activation cluster (SMAC) enriched for TCR/CD3 complex (cSMAC, light green), with a outer ring of LFA-1/ICAM-1 (pink). The zone between cSMAC and pSMAC is depicted (deep pink), where high-affinity LFA-1–ICAM-1 interaction occurs with intense localization of RAP1 and Kindlin-3. MST1 activation stimulated by RAP1 and RAPL relocates vesicular transport factors important for SMAC formation including TCR and LFA-1. Possible connections between MST1/NDR1 and downstream regulators for the maturation of IS are proposed. Components of ESCRT such as TSG101 and VPS4 are required for the release of TCR-containing microvesicles (TCR vesicles) and cSMAC maturation. After internalization of TCR-containing vesicles, IFT20 recognizes them and relays them to the Rab11^+^ recycling endosome, which contains RAB35 and RAB29. Subsequently, RAB8, an important factor of vesicular transport in polarized cells, is replaced by RAB11 in TCR-containing vesicle to promote membrane targeting and repositioning.

At the antigen recognition on the APC, lymphocytes undergo reorganization of organelles and transport antigen receptor and integrin to contact site in order to form IS, which is an important platform for signal transduction during the initiation of lymphocyte activation and polarized release of cytotoxic granules (80–84). MST1/2 play critical roles in mature IS formation, highlighting their important roles in redistribution of membrane receptors and organelles. The IS is composed mainly of four layers of supramolecular activation cluster (SMAC) as shown in Figure 4. Upon IS maturation, TCR is accumulated at central SMAC (cSMAC) area, whereas LFA-1/ICAM-1 clusters surround cSMAC to form peripheral SMAC (pSMAC) with ring structure (Figure 4). MST1 localizes mainly at pSMAC area of IS (34). MST1-deficiency or MST1/2-deficiency cause defective adhesion and pSMAC formation due to the loss of accumulation and long-term binding between LFA-1 and ICAM-1. This defect results from a failure of Kindlin-3 recruitment to the cSMAC-pSMAC border area, where high-affinity binding between LFA-1 and ICAM-1 occurs (34) (Figure 4). Furthermore, the absence of MST1/2 or introduction of a kinase-dead MST1 mutant in T cells impairs not only pSMAC but also cSMAC formation, indicating that MST1/2 kinase activities are also required for relocation of TCR as well as LFA-1. Recent studies show that cSMAC formation is facilitated by vesicular transport of TCR-enriched microvesicles, which are derived from endosomal sorting complexes required for transport (ESCRT) and endosome recycling pathways (Figure 4). MST1/2 deficiency cause low levels of accumulation of ESCRT and endosome recycling regulators, such as VPS4, RAB8, RAB11, in the vicinity of the contact plane during the IS formation (34) (Figure 4). These results point out the important roles of MST1/2 in vesicular transport in lymphocytes.

In line with this notion, several groups have reported the role of MST1 in polarized trafficking of LFA-1 through Rab family small GTPases by activating guanine exchange factor (GEF). One study reported that MST1 phosphorylates DENDD1C, a GEF of RAB13, to activate and recruit RAB13 to the leading edge, thereby facilitating LFA-1 transport to the front (85). In neutrophils, MST1 regulates translocation of RAB27 to control transport of integrins and neutrophil elastase probably through the regulation of GEF JFC-1 (86). Thus, MST1/2 play a key role in polarized transport of both LFA-1 and TCR (Figure 4).

Moreover, NDR1/2 are involved in the IS formation and regulation of vesicle trafficking downstream of MST1/2. *Ndr1* knockdown causes defects in IS formation and impairs recruitment of Rab family proteins at the contact surface, similar to MST1/2 deficiency (34). The mechanism by which NDR1 regulates vesicle trafficking is still unclear. NDR1 would control vesicle trafficking through Rho family small GTPases as described above. NDR1-dependent regulation of Kindlin-3 suggests that integrin-mediated signaling also contributes to spatiotemporal activation of Rac- and Rho-GTPases, thereby providing a positional cue for reorganization of vesicle transport machinery. Alternatively, NDR1/2 could directly regulate Rab family small GTPases in lymphocytes. In neurons, the phosphorylation targets of NDR1/2 have been identified using a chemical genetics approach (13). Most of the identified proteins are related to vesicular transport machinery, e.g., AP2-associated kinase (AAK1), RAB3AIP–RAB3A interacting protein (RABIN8), and Rab11 family interacting protein 5 (RAB11FIP5). Indeed, they contribute to the regulation of neurons and other cells via NDR1/2 dependent vesicular transport (13). Therefore, it is possible that NDR1/2 directly activate these factors to trigger vesicular trafficking in lymphocytes as well (Figure 4).

Collectively, MST1/2 and NDR1/2 play key roles for polarized transport using the vesicular trafficking machinery for both integrin and antigen receptor trafficking and control IS formation (Figure 4); however, further studies are warranted to elucidate the complexity of uncharacterized mechanisms of MST1/2-dependent vesicular transport of integrin and TCR.

In summary, the studies described above strongly suggest that MST1/2 are directly involved in the regulation of integrin activation, cytoskeleton dynamics, and the vesicular transport machineries. These processes are tightly associated with cell adhesion behaviors and must be coordinately regulated in lymphocyte trafficking and antigen responses. We speculate that MST1/2 associate with several types of intracellular trafficking vesicles, such as early/late/recycling endosomes and exocytic vesicles, and phosphorylate distinct effector molecules *in situ* to drive step-wise activation and recruitment of final effector molecules, such as integrin and F-actin. The details of the interplay of these processes downstream of MST1/2 will reveal exciting new mechanisms and functions of Hippo signaling.

## Conclusion

Accumulating evidence indicates that MST1/2 have multiple functions, including lymphocyte trafficking, effector differentiation and functions, and tolerance. These immune functions, mostly mediated by pathways distinct from the canonical Hippo pathway, involve regulation of integrin-mediated adhesion, cell polarity and transcription factors. These downstream intercellular signaling events are elicited by spatiotemporal regulation of the kinase activity of MST1/2, leading to transcriptional control of gene expressions and vesicle trafficking pathways. Studies of these processes have established a new framework for Hippo signaling in immune homeostasis and diseases, and should lead to the development of therapeutic strategies to control these processes.

## Author Contributions

YU organized the manuscript. YU and NK wrote the draft and prepared the figures. YU, NK, and TK revised the draft. YU and TK edited the language and figures.

## Conflict of Interest

The authors declare that the research was conducted in the absence of any commercial or financial relationships that could be construed as a potential conflict of interest.

## References

[1] Harvey KF Pfleger CM Hariharan IK. The *Drosophila* Mst ortholog, hippo, restricts growth and cell proliferation and promotes apoptosis. *Cell.* (2003) 114:457–67. 10.1016/s0092-867400557-9 12941274

[2] Pantalacci S Tapon N L P. Éopold: the salvador partner hippo promotes apoptosis and cell-cycle exit in *Drosophila*. *Nat Cell Biol.* (2003) 5:921–7. 10.1038/ncb1051 14502295

[3] Udan RS Kango-Singh M Nolo R Tao C Halder G. Hippo promotes proliferation arrest and apoptosis in the Salvador/Warts pathway. *Nat Cell Biol.* (2003) 5:914–20. 10.1038/ncb1050 14502294

[4] Wu S Huang J Dong J Pan D. hippo encodes a Ste-20 family protein kinase that restricts cell proliferation and promotes apoptosis in conjunction with salvador and warts. *Cell.* (2003) 114:445–56. 10.1016/s0092-867400549-x 12941273

[5] Koike-Kumagai M Yasunaga K Morikawa R Kanamori T Emoto K. The target of rapamycin complex 2 controls dendritic tiling of Drosophila sensory neurons through the Tricornered kinase signalling pathway. *EMBO J.* (2009) 28:3879–92. 10.1038/emboj.2009.312 19875983 PMC2797056

[6] Emoto K Parrish JZ Jan LY Jan YN. The tumour suppressor Hippo acts with the NDR kinases in dendritic tiling and maintenance. *Nature.* (2006) 443:210–3. 10.1038/nature05090 16906135

[7] Geng W He B Wang M Adler PN. The tricornered gene, which is required for the integrity of epidermal cell extensions, encodes the *Drosophila* nuclear DBF2-related kinase. *Genetics.* (2000) 156:1817–28. 11102376 10.1093/genetics/156.4.1817 PMC1461384

[8] Zhou D Conrad C Xia F Park JS Payer B Yin Y Mst1 and Mst2 maintain hepatocyte quiescence and suppress hepatocellular carcinoma development through inactivation of the Yap1 oncogene. *Cancer Cell.* (2009) 16:425–38. 10.1016/j.ccr.2009.09.026 19878874 PMC3023165

[9] Zhou D Zhang Y Wu H Barry E Yin Y Lawrence E Mst1 and Mst2 protein kinases restrain intestinal stem cell proliferation and colonic tumorigenesis by inhibition of Yes-associated protein (Yap) overabundance. *Proc Natl Acad Sci USA.* (2011) 108:E1312–20. 10.1073/pnas.1110428108 22042863 PMC3241824

[10] Vichalkovski A Gresko E Cornils H Hergovich A Schmitz D Hemmings BA. NDR kinase is activated by RASSF1A/MST1 in response to Fas receptor stimulation and promotes apoptosis. *Curr Biol.* (2008) 18:1889–95. 10.1016/j.cub.2008.10.060 19062280

[11] Hergovich A Kohler RS Schmitz D Vichalkovski A Cornils H Hemmings BA. The MST1 and hMOB1 tumor suppressors control human centrosome duplication by regulating NDR kinase phosphorylation. *Curr Biol.* (2009) 19:1692–702. 10.1016/j.cub.2009.09.020 19836237

[12] Zhang L Tang F Terracciano L Hynx D Kohler R Bichet S NDR functions as a physiological YAP1 kinase in the intestinal epithelium. *Curr Bio.* (2015) 25:296–305. 10.1016/j.cub.2014.11.054 25601544 PMC4426889

[13] Ultanir SK Hertz NT Li G Ge WP Burlingame AL Pleasure SJ Chemical genetic identification of NDR1/2 kinase substrates AAK1 and Rabin8 Uncovers their roles in dendrite arborization and spine development. *Neuron.* (2012) 73:1127–42. 10.1016/j.neuron.2012.01.019 22445341 PMC3333840

[14] Abdollahpour H Appaswamy G Kotlarz D Diestelhorst J Beier R Schäffer AA The phenotype of human STK4 deficiency. *Blood.* (2012) 119:3450–7. 10.1182/blood-2011-09-378158 22294732 PMC3325036

[15] Crequer A Picard C Patin E D’Amico A Abhyankar A Munzer M Inherited MST1 deficiency underlies susceptibility to EV-HPV infections. *PLoS One.* (2012) 7:e44010. 10.1371/journal.pone.0044010 22952854 PMC3428299

[16] Dang TS Willet JD Griffin HR Morgan NV O’Boyle G Arkwright PD Defective leukocyte adhesion and chemotaxis contributes to combined immunodeficiency in humans with autosomal recessive MST1 deficiency. *J Clin Immunol.* (2016) 36:117–22. 10.1007/s10875-016-0232-2 26801501 PMC4769310

[17] Nehme NT Pachlopnik Schmid J Debeurme F André-Schmutz I Lim A Nitschke P MST1 mutations in autosomal recessive primary immunodeficiency characterized by defective naive T-cell survival. *Blood.* (2012) 119:3458–68. 10.1182/blood-2011-09-378364 22174160 PMC3824282

[18] Ueda Y Katagiri K Tomiyama T Yasuda K Habiro K Katakai T Mst1 regulates integrin-dependent thymocyte trafficking and antigen recognition in the thymus. *Nat Commun.* (2012) 3:1098. 10.1038/ncomms2105 23033074

[19] Dong Y Du X Ye J Han M Xu T Zhuang Y A cell-intrinsic role for Mst1 in regulating thymocyte egress. *J Immunol.* (2009) 183:3865–72. 10.4049/jimmunol.0900678 19692642

[20] Choi J Oh S Lee D Oh HJ Park JY Lee SB Mst1-FoxO signaling protects Naive T lymphocytes from cellular oxidative stress in mice. *PLoS One.* (2009) 4:e8011. 10.1371/journal.pone.0008011 19956688 PMC2776980

[21] Katagiri K Katakai T Ebisuno Y Ueda Y Okada T Kinashi T. Mst1 controls lymphocyte trafficking and interstitial motility within lymph nodes. *EMBO J.* (2009) 28:1319–31. 10.1038/emboj.2009.82 19339990 PMC2683056

[22] Zhou D Medoff BD Chen L Li L Zhang XF Praskova M vruch: The Nore1B/Mst1 complex restrains antigen receptor-induced proliferation of naïve T cells. *Proc Natl Acad Sci USA.* (2008) 105:20321–6. 10.1073/pnas.0810773105 19073936 PMC2600581

[23] Du X Shi H Li J Dong Y Liang J Ye J Mst1/Mst2 regulate development and function of regulatory T cells through modulation of Foxo1/Foxo3 stability in autoimmune disease. *J Immunol.* (2014) 192:1525–35. 10.4049/jimmunol.1301060 24453252

[24] Kawabata K Ujikawa M Egawa T Kawamoto H Tachibana K Iizasa H A cell-autonomous requirement for CXCR4 in long-term lymphoid and myeloid reconstitution. *Proc Natl Acad Sci USA.* (1999) 96:5663–7. 10.1073/pnas.96.10.5663 10318941 PMC21917

[25] Szilvassy SJ Meyerrose TE Ragland PL Grimes B. Differential homing and engraftment properties of hematopoietic progenitor cells from murine bone marrow, mobilized peripheral blood, and fetal liver. *Blood.* (2001) 98:2108–15. 10.1182/blood.v98.7.2108 11567997

[26] Lee DH Kim TS Lee D Lim DS. Mammalian sterile 20 kinase 1 and 2 are important regulators of hematopoietic stem cells in stress condition. *Sci Rep.* (2018) 8:942. 10.1038/s41598-018-19637-y 29343826 PMC5772645

[27] Ruiz P Wiles MV Imhof BA. Alpha 6 integrins participate in pro-T cell homing to the thymus. *Eur J Immunol.* (1995) 25:2034–41. 10.1002/eji.1830250735 7621877

[28] Scimone ML Aifantis I Apostolou I von Boehmer H von Andrian UH. A multistep adhesion cascade for lymphoid progenitor cell homing to the thymus. *Proc Natl Acad Sci USA.* (2006) 103:7006–11. 10.1073/pnas.0602024103 16641096 PMC1459009

[29] Moretti FA Klapproth S Ruppert R Margraf A Weber J Pick R Differential requirement of kindlin-3 for T cell progenitor homing to the non-vascularized and vascularized thymus. *eLife.* (2018) 7:35816. 10.7554/eLife.35816 30187863 PMC6126919

[30] Love PE Bhandoola A. Signal integration and crosstalk during thymocyte migration and emigration. *Nat Rev Immunol.* (2011) 11:469–77. 10.1038/nri2989 21701522 PMC3710714

[31] Anderson MS Venanzi ES Klein L Chen Z Berzins SP Turley SJ Projection of an immunological self shadow within the thymus by the aire protein. *Science.* (2002) 298:1395–401. 10.1126/science.1075958 12376594

[32] Le Borgne M Ladi E Dzhagalov I Herzmark P Liao YF Chakraborty AK The impact of negative selection on thymocyte migration in the medulla. *Nat Immunol.* (2009) 10:823–30. 10.1038/ni.1761 19543275 PMC2793676

[33] Mou F Praskova M Xia F Van Buren D Hock H Avruch J The Mst1 and Mst2 kinases control activation of rho family GTPases and thymic egress of mature thymocytes. *J Exp Med.* (2012) 209:741–59. 10.1084/jem.20111692 22412158 PMC3328371

[34] Kondo N Ueda Y Kita T Ozawa M Tomiyama T Yasuda K NDR1-Dependent regulation of kindlin-3 controls high-affinity LFA-1 binding and immune synapse organization. *Mol Cell Biol.* (2017) 37:424–416. 10.1128/mcb.00424-16 28137909 PMC5376635

[35] Allman D Pillai S. Peripheral B cell subsets. *Curr Opin Immunol.* (2008) 20:149–57. 10.1016/j.coi.2008.03.014 18434123 PMC2532490

[36] Alsufyani F Mattoo H Zhou D Cariappa A Van Buren D Hock H The Mst1 kinase is required for follicular B cell homing and B-1 B cell development. *Front Immunol.* (2018) 9:2393. 10.3389/fimmu.2018.02393 30386341 PMC6199389

[37] Wang H Beaty N Chen S Qi CF Masiuk M Shin DM The CXCR7 chemokine receptor promotes B-cell retention in the splenic marginal zone and serves as a sink for CXCL12. *Blood.* (2012) 119:465–8. 10.1182/blood-2011-03-343608 22110250 PMC3257011

[38] Cinamon G Matloubian M Lesneski MJ Xu Y Low C Lu T Sphingosine 1-phosphate receptor 1 promotes B cell localization in the splenic marginal zone. *Nat Immunol.* (2004) 5:713–20. 10.1038/ni1083 15184895

[39] Lu TT Cyster JG. Integrin-mediated long-term B cell retention in the splenic marginal zone. *Science.* (2002) 297:409–12. 10.1126/science.1071632 12130787

[40] Manevich-Mendelson E Grabovsky V Feigelson SW Cinamon G Gore Y Goverse G Talin1 is required for integrin-dependent B lymphocyte homing to lymph nodes and the bone marrow but not for follicular B-cell maturation in the spleen. *Blood.* (2010) 116:5907–18. 10.1182/blood-2010-06-293506 20923969

[41] Okada T Ngo VN Ekland EH Forster R Lipp M Littman DR Chemokine requirements for B cell entry to lymph nodes and Peyer’s patches. *J Exp Med.* (2002) 196:65–75. 10.1084/jem.20020201 12093871 PMC2194009

[42] Salojin KV Hamman BD Chang WC Jhaver KG Al-Shami A Crisostomo J Genetic deletion of Mst1 alters T cell function and protects against autoimmunity. *PLoS One.* (2014) 9:e98151. 10.1371/journal.pone.0098151 24852423 PMC4031148

[43] Tomiyama T Ueda Y Katakai T Kondo N Okazaki K Kinashi T. Antigen-specific suppression and immunological synapse formation by regulatory T cells require the Mst1 kinase. *PLoS One.* (2013) 8:e73874. 10.1371/journal.pone.0073874 24040101 PMC3767606

[44] Onishi Y Fehervari Z Yamaguchi T Sakaguchi S. Foxp3+ natural regulatory T cells preferentially form aggregates on dendritic cells in vitro and actively inhibit their maturation. *Proc Natl Acad Sci USA.* (2008) 105:10113–8. 10.1073/pnas.0711106105 18635688 PMC2481354

[45] Kerdiles YM Stone EL Beisner DR Beisner DL Chen IL Stockmann C Foxo transcription factors control regulatory T cell development and function. *Immunity.* (2010) 33:890–904. 10.1016/j.immuni.2010.12.002 21167754 PMC3034255

[46] Ouyang W Beckett O Ma Q Paik JH DePinho RA Li MO. Foxo proteins cooperatively control the differentiation of Foxp3+ regulatory T cells. *Nat Immunol.* (2010) 11:618–27. 10.1038/ni.1884 20467422

[47] van Loosdregt J Vercoulen Y Guichelaar T Gent YY Beekman JM van Beekum O Regulation of Treg functionality by acetylation-mediated Foxp3 protein stabilization. *Blood.* (2010) 115:965–74. 10.1182/blood-2009-02-207118 19996091

[48] Li J Du X Shi H Deng K Chi H Tao W. Mammalian sterile 20-like kinase 1 (Mst1) enhances the stability of forkhead box P3 (Foxp3) and the function of regulatory T cells by modulating Foxp3 acetylation. *J Biol Chem.* (2015) 290:30762–70. 10.1074/jbc.M115.668442 26538561 PMC4692206

[49] Yuan F Xie Q Wu J Bai Y Mao B Dong Y MST1 promotes apoptosis through regulating Sirt1-dependent p53 deacetylation. *J Biol Chem.* (2011) 286:6940–5. 10.1074/jbc.M110.182543 21212262 PMC3044949

[50] Shi H Liu C Tan H Li Y Nguyen TM Dhungana Y Hippo Kinases Mst1 and Mst2 Sense and Amplify IL-2R-STAT5 signaling in regulatory T cells to establish stable regulatory activity. *Immunity.* (2018) 49:899–914. 10.1016/j.immuni.2018.10.010 30413360 PMC6249059

[51] Yasuda K Ueda Y Ozawa M Matsuda T Kinashi T. Enhanced cytotoxic T-cell function and inhibition of tumor progression by Mst1 deficiency. *FEBS Lett.* (2016) 590:68–75. 10.1002/1873-3468.12045 26787462

[52] Thaventhiran JE Hoffmann A Magiera L de la Roche M Lingel H Brunner-Weinzierl M Activation of the Hippo pathway by CTLA-4 regulates the expression of Blimp-1 in the CD8+ T cell. *Proc Natl Acad Sci USA.* (2012) 109:E2223–9. 10.1073/pnas.1209115109 22745171 PMC3421161

[53] Meng Z Moroishi T Mottier-Pavie V Plouffe SW Hansen CG Hong AW MAP4K family kinases act in parallel to MST1/2 to activate LATS1/2 in the Hippo pathway. *Nat Commun.* (2015) 6:8357. 10.1038/ncomms9357 26437443 PMC4600732

[54] Geng J Yu S Zhao H Sun X Li X Wang P The transcriptional coactivator TAZ regulates reciprocal differentiation of T. *Nat Immunol.* (2017) 18:800–12. 10.1038/ni.3748 28504697

[55] Park E Kim MS Song JH Roh KH Lee R Kim TS. MST1 deficiency promotes B cell responses by CD4. *Biochem Biophys Res Commun.* (2017) 489:56–62. 10.1016/j.bbrc.2017.05.094 28527887

[56] Yamamura K Uruno T Shiraishi A Tanaka Y Ushijima M Nakahara T The transcription factor EPAS1 links DOCK8 deficiency to atopic skin inflammation via IL-31 induction. *Nat Commun.* (2017) 8:13946. 10.1038/ncomms13946 28067314 PMC5228069

[57] Lehtinen MK Yuan Z Boag PR Yang Y Villén J Becker EB A conserved MST-FOXO signaling pathway mediates oxidative-stress responses and extends life span. *Cell.* (2006) 125:987–1001. 10.1016/j.cell.2006.03.046 16751106

[58] Hagenbuchner J Ausserlechner MJ. Mitochondria and FOXO3: breath or die. *Front Physiol.* (2013) 4:147 10.3389/fphys.2013.00147 PMC3687139 23801966

[59] Klann JE Kim SH Remedios KA He Z Metz PJ Lopez J Integrin Activation controls regulatory T cell-mediated peripheral tolerance. *J Immunol.* (2018) 200:4012–23. 10.4049/jimmunol.1800112 29703862 PMC5988969

[60] Klann JE Remedios KA Kim SH Metz PJ Lopez J Mack LA Talin Plays a critical role in the maintenance of the regulatory T cell pool. *J Immuno.* (2017) 198:4639–51. 10.4049/jimmunol.1601165 PMC5507362 28515282

[61] Haasken S Auger JL Binstadt BA. Absence of β2 integrins impairs regulatory T cells and exacerbates CD4+ T cell-dependent autoimmune carditis. *J Immunol.* (2011) 187:2702–10. 10.4049/jimmunol.1000967 21795599 PMC3159859

[62] Li L Kim J Boussiotis VA. Rap1A regulates generation of T regulatory cells via LFA-1-dependent and LFA-1-independent mechanisms. *Cell Immunol.* (2010) 266:7–13. 10.1016/j.cellimm.2010.08.014 20864093 PMC2966523

[63] Tang F Gill J Ficht X Barthlott T Cornils H Schmitz-Rohmer D The kinases NDR1/2 act downstream of the Hippo homolog MST1 to mediate both egress of thymocytes from the thymus and lymphocyte motility. *Sci Signal.* (2015) 8:ra100 10.1126/scisignal.aab2425 26443704

[64] Katagiri K Maeda A Shimonaka M Kinashi T. RAPL, a Rap1-binding molecule that mediates Rap1-induced adhesion through spatial regulation of LFA-1. *Nat Immunol.* (2003) 4:741–8. 10.1038/ni950 12845325

[65] Katagiri K Imamura M Kinashi T. Spatiotemporal regulation of the kinase Mst1 by binding protein RAPL is critical for lymphocyte polarity and adhesion. *Nat Immunol.* (2006) 7:919–28. 10.1038/ni1374 16892067

[66] Wynne JP Wu J Su W Mor A Patsoukis N Boussiotis VA Rap1-interacting adapter molecule (RIAM) associates with the plasma membrane via a proximity detector. *J Cell Biol.* (2012) 199:317–30. 10.1083/jcb.201201157 23045549 PMC3471229

[67] Kliche S Worbs T Wang X Degen J Patzak I Meineke B CCR7-mediated LFA-1 functions in T cells are regulated by 2 independent ADAP/SKAP55 modules. *Blood.* (2012) 119:777–85. 10.1182/blood-2011-06-362269 22117043

[68] Raab M Wang H Lu Y Smith X Wu Z Strebhardt K T cell receptor “inside-out” pathway via signaling module SKAP1-RapL regulates T cell motility and interactions in lymph nodes. *Immunity.* (2010) 32:541–56. 10.1016/j.immuni.2010.03.007 20346707 PMC3812847

[69] Okada T Sinha S Esposito I Schiavon G López-Lago MA Su W The Rho GTPase Rnd1 suppresses mammary tumorigenesis and EMT by restraining Ras-MAPK signalling. *Nat Cell Biol.* (2015) 17:81–94. 10.1038/ncb3082 25531777 PMC4374353

[70] Klapproth S Sperandio M Pinheiro EM Prünster M Soehnlein O Gertler FB Loss of the Rap1 effector RIAM results in leukocyte adhesion deficiency due to impaired β2 integrin function in mice. *Blood.* (2015) 126:2704–12. 10.1182/blood-2015-05-647453 26337492 PMC4683331

[71] Zhu L Yang J Bromberger T Holly A Lu F Liu H Structure of Rap1b bound to talin reveals a pathway for triggering integrin activation. *Nat Commun.* (2017) 8:1744. 10.1038/s41467-017-01822-8 29170462 PMC5701058

[72] Bromberger T Klapproth S Rohwedder I Zhu L Mittmann L Reichel CA Direct Rap1/Talin1 interaction regulates platelet and neutrophil integrin activity in mice. *Blood.* (2018) 132:2754–62. 10.1182/blood-2018-04-846766 30442677 PMC6307989

[73] Gingras AR Lagarrigue F Cuevas MN Valadez AJ Zorovich M McLaughlin W Rap1 binding and a lipid-dependent helix in talin F1 domain promote integrin activation in tandem. *J Cell Biol.* (2019) 218:1799–809. 10.1083/jcb.201810061 30988001 PMC6548133

[74] Waldt N Seifert A Demiray YE Devroe E Turk BE Reichardt P Filamin a phosphorylation at serine 2152 by the serine/threonine kinase Ndr2 controls TCR-induced LFA-1 activation in T cells. *Front Immunol.* (2018) 9:2852. 10.3389/fimmu.2018.02852 30568657 PMC6290345

[75] Kato W Nishio M To Y Togashi H Mak TW Takada H MOB1 regulates thymocyte egress and T-cell survival in mice in a YAP1-independent manner. *Genes Cells.* (2019) 24:485–95. 10.1111/gtc.12704 31125466

[76] Katagiri K Ohnishi N Kabashima K Iyoda T Takeda N Shinkai Y Crucial functions of the Rap1 effector molecule RAPL in lymphocyte and dendritic cell trafficking. *Nat Immunol.* (2004) 5:1045–51. 10.1038/ni1111 15361866

[77] Xu X Wang X Todd EM Jaeger ER Vella JL Mooren OL Mst1 Kinase regulates the actin-bundling protein L-plastin to promote T cell migration. *J Immunol.* (2016) 197:1683–91. 10.4049/jimmunol.1600874 27465533 PMC4992580

[78] Lee JH Katakai T Hara T Gonda H Sugai M Shimizu A. Roles of p-ERM and Rho-ROCK signaling in lymphocyte polarity and uropod formation. *J Cell Biol.* (2004) 167:327–37. 10.1083/jcb.200403091 15504914 PMC2172551

[79] Xu X Jaeger ER Wang X Lagler-Ferrez E Batalov S Mathis NL Mst1 directs Myosin IIa partitioning of low and higher affinity integrins during T cell migration. *PLoS One.* (2014) 9:e105561. 10.1371/journal.pone.0105561 25133611 PMC4136924

[80] Martin-Cofreces NB Baixauli F Sanchez-Madrid F. Immune synapse: conductor of orchestrated organelle movement. *Trends Cell Biol.* (2014) 24:61–72. 10.1016/j.tcb.2013.09.005 24119664 PMC4347664

[81] Dupre L Houmadi R Tang C Rey-Barroso J. T lymphocyte migration: an action movie starring the actin and associated actors. *Front Immunol.* (2015) 6:586. 10.3389/fimmu.2015.00586 26635800 PMC4649030

[82] Krummel MF Bartumeus F Gerard A. T cell migration, search strategies and mechanisms. *Nat Rev Immunol.* (2016) 16:193–201. 10.1038/nri.2015.16 26852928 PMC4869523

[83] Kabanova A Zurli V Baldari CT. Signals controlling lytic granule polarization at the cytotoxic immune synapse. *Front Immunol.* (2018) 9:307. 10.3389/fimmu.2018.00307 29515593 PMC5826174

[84] Tsun A Qureshi I Stinchcombe JC Jenkins MR de la Roche M Kleczkowska J Centrosome docking at the immunological synapse is controlled by Lck signaling. *J Cell Biol.* (2011) 192:663–74. 10.1083/jcb.201008140 21339332 PMC3044125

[85] Nishikimi A Ishihara S Ozawa M Etoh K Fukuda M Kinashi T Rab13 acts downstream of the kinase Mst1 to deliver the integrin LFA-1 to the cell surface for lymphocyte trafficking. *Sci Signal.* (2014) 7:ra72 10.1126/scisignal.2005199 25074980

[86] Kurz AR Pruenster M Rohwedder I Ramadass M Schäfer K Harrison U MST1-dependent vesicle trafficking regulates neutrophil transmigration through the vascular basement membrane. *J Clin Invest.* (2016) 126:4125–39. 10.1172/JCI87043 27701149 PMC5096904

